# Single‐cell transcriptome analysis of male chicken germ cells reveals changes in signaling pathway‐related gene expression profiles during mitotic arrest

**DOI:** 10.1002/2211-5463.13600

**Published:** 2023-03-30

**Authors:** Hyeon Jeong Choi, Kyung Min Jung, Kyung Je Park, Kyung Youn Lee, Seung Je Woo, Jae Yong Han

**Affiliations:** ^1^ Biomodulation Major, Department of Agricultural Biotechnology and Research Institute of Agriculture and Life Sciences Seoul National University South Korea

**Keywords:** chicken, male germ cell, mitotic arrest, signaling pathway, single‐cell RNA sequencing

## Abstract

Mitotic arrest is necessary for the embryonic development of germ cells, and thus, it is important to understand the signaling pathways that regulate mitotic arrest. Here, we investigated the signaling pathway dynamics of male embryonic chicken germ cells during mitotic arrest by single‐cell transcriptome analysis using germ‐cell tracing models. We identified signaling pathways that change at the transcriptional level during chicken male germ‐cell development after sex determination. We found that several components of the BMP, Notch, and JAK–STAT signaling pathways were downregulated at the mitotic‐arrest stage and were reactivated 1 week after hatching when all germ cells are quiescent after entering mitotic arrest. In addition, the transcriptional levels of components of the MAPK, Hedgehog, and thyroid‐hormone signaling pathways were steadily upregulated after mitotic arrest. This suggests the cooperation of multiple signaling pathways during entry into mitotic arrest and subsequent quiescence of chicken male germ cells.

## Graphical abstract

We identified the signaling pathway dynamics of male embryonic chicken germ cells during mitotic arrest by single‐cell transcriptome analysis of germ cells isolated from a germ‐cell tracing chicken model (*DAZL*::*GFP* chicken). BMP, Notch, and JAK–STAT signaling were downregulated at the mitotic‐arrest stage and were reactivated 1 week after hatching. On the contrary, MAPK, Hedgehog, and thyroid‐hormone (TH) signaling steadily increased after mitotic arrest.

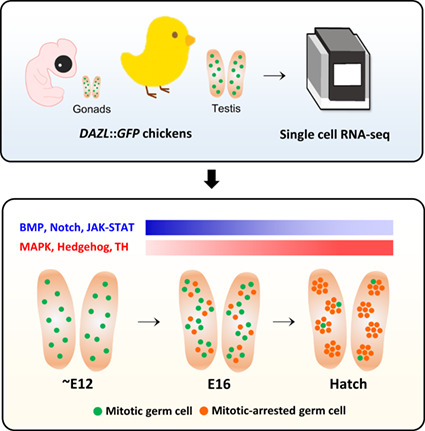

**Abbreviations**
BMP: bone morphogenetic protein
DEG: differentially expressed genes
FACS: fluorescence‐activated cell sorting
FDR: false discovery rate
FGF: fibroblast growth factor
GO: gene ontology
JAK–STAT: Janus kinases‐signal transducer and activator of transcription proteins
MAPK: mitogen‐activated protein kinase
PGC: primordial germ cell
PI: propidium iodide
qRT‐PCR: quantitative RT‐PCR
scRNA‐seq: single‐cell RNA‐sequencing
TGF‐β: transforming growth factor‐beta
UMAP: uniform manifold approximation and projection

Mitotic arrest is a unique essential event that occurs during the development of male germ cells from the embryonic stage into a spermatocyte pool [1, 2, 3]. In chickens, during the differentiation of primordial gem cells (PGCs) into sex‐specific germ cells, female germ cells enter meiosis and maintain meiotic arrest in G2/M phase until hatching, whereas male germ cells asynchronously enter mitotic arrest on embryonic day 14 (E14) and then enter into the resting phase (mitotic quiescence state). At 10 weeks after hatching, they re‐enter the cell cycle and begin to differentiate [3, 4, 5]. The mitotic arrest in germ cells is regulated by multiple signaling pathways in diverse species [6, 7, 8, 9, 10]. Previously signaling pathways involved in differentiating germ cells were analyzed at different stages of chicken embryo development by genome and transcriptome‐wide analyses after their separation from somatic cells. This approach revealed that signaling pathways such as TGF‐β, JAK–STAT, and Hedgehog are involved in chicken germ‐cell mitotic arrest [11]. However, we have yet to understand the pathways that regulate male germ‐cell‐specific differentiation, including mitotic arrest, during *in vivo* development.

Single‐cell RNA sequencing (scRNA‐seq) has enabled the study of the signaling pathways responsible for germ‐cell mitotic arrest in several species. In humans, fetal male germ cells enter mitotic arrest 9 weeks after fertilization, and TGF‐β‐pathway‐related genes including those related to the BMP and NODAL pathways are highly expressed in mitotically arrested fetal germ cells. Moreover, modulation of the expression of some ligands *AMH* and *NODAL* may be important for germ‐cell mitotic arrest [10]. In mice, Notch signaling is more active in PGCs transitioning to mitotic arrest, and blocking the expression of *HES1* and *NOTCH1* belonging to the Notch signaling prevents PGCs from properly entering mitotic arrest [9]. Hence, Notch signaling may initiate mitotic arrest in mouse male germ cells and may be essential for maintaining germ‐cell identity. Also, in mice, Hippo signaling is transiently more active 2 days after birth when male germ cells exit mitotic arrest [12].

Recently, we developed germ‐cell tracing chicken models expressing germ‐cell‐specific fluorescent markers and were able to perform scRNA‐seq of germ cells at each stage of embryonic development using single‐cell transcriptomic approach [13]. Using this model, we identified the mitotic‐arrested prospermatogonia cluster at three time points (E12, E16, and hatch) and found an epigenetic reprogramming schedule of mitotic‐arrested chicken prospermatogonia [5]. As a follow‐up study, here we investigate signaling pathways that regulate male embryonic germ cells during mitotic arrest for the first time using a single‐cell transcriptomic approach. We found dynamic changes in multiple signaling pathways during mitotic arrest of male embryonic chicken germ cells, which were also observed when the developmental stage was extended beyond hatching. It is proposed that multiple pathways whose activities change before and after mitotic arrest cooperatively regulate mitotic arrest and subsequent quiescent state of male germ cells in chickens.

## Materials and methods

### Experimental animals and animal care

All experimental procedures and care of chickens were approved by the Institute of Laboratory Animal Resources, Seoul National University. All methods were performed in accordance with ARRIVE (Animal Research: Reporting of *In Vivo* Experiments) guidelines and approved by the Institutional Animal Care and Use Committee (IACUC, SNU‐190401‐1‐3) of Seoul National University.

### Single‐cell library preparation and sequencing

To isolate stage‐specific germ cells, we used previously developed germ‐cell tracing chicken models that express a germ‐cell‐specific fluorescent marker (GFP) [13]. DAZL::GFP germ cells at E2.5, E6, E8, E12, E16, hatch and 1‐week posthatch were collected by using a fluorescence‐activated cell sorting (FACS) Aria III (BD Biosciences, San Jose, CA, USA). To isolate live cells, cells were stained with propidium iodide (PI), and GFP^+^/PI^−^ germ cells were sorted. Libraries for scRNA‐seq were prepared by using the Chromium Single Cell 3′ GEM, Library & Gel Bead Kit v3 (PN‐1000075, 10× Genomics, Pleasanton, CA, USA); Chromium Single Cell B‐Chip Kit (PN‐1000073, 10× Genomics); and Chromium i7 Multiplex Kit (PN‐120262, 10× Genomics). Libraries were sequenced with a 2 × 100 bp paired‐end protocol on a Novaseq‐6000 platform (Illumina, San Diego, CA, USA). Detailed information is shown in our previous studies [5, 13].

### Single‐cell RNA‐seq data processing and analysis

Raw fastq files were processed using the cellranger pipeline, version 3.1.0 (10x Genomics, Pleasanton, CA, USA). The fasta and GTF files for chicken genome (GRCg6a) were modified to include the *DAZL*::*GFP* insert sequence. The cDNA sequences were mapped to the modified‐chicken genome by using star (version 2.5.1b) aligner (GitHub, Inc., San Francisco, CA, USA) with the GRCg6a.99 GTF file. After aggregating gene‐by‐cell count matrixes of E12, E16, and hatch to remove cell‐specific biases, cells were clustered by using the quickCluster function of the scran (version 1.16.0) r package (Bioconductor, Boston, MA, USA) [14]. Differentially expressed genes (DEGs) among five clusters (clusters 1, 2, 3, 4, and 5) were identified by using the FindMarkers function of the seurat r package (FDR < 0.05). Gene Ontology (GO) terms enrichment analysis in DEGs (FDR < 0.05) of cluster 1 was performed using PANTHER [15], and ‘GO biological process complete’ was the annotation dataset. Among these, terms with fewer than three included genes or terms with associated *P*‐values ≥ 1 were excluded. Count matrixes of E2.5 to 1 week after hatch were further aggregated and re‐normalized with scran r package. Detailed information is shown in our previous studies [5].

### Immunohistochemistry

Immunohistochemistry was performed to identify the expression of GFP and DAZL in germ cells of *DAZL*::*GFP* chick testis. Testes of chicks at hatching day were paraffin‐embedded and sectioned (thickness, 9–10 μm). After deparaffinization, sections were washed three times with PBS and blocked with a blocking buffer (5% goat serum and 1% bovine serum albumin in PBS) for 1 h at room temperature. Sections were then incubated at 4 °C overnight with primary antibodies, rabbit anti‐GFP (Invitrogen, Thermo Fisher Scientific Inc., Carlsbad, CA, USA), or rabbit anti‐DAZL [16]. After washing three times with PBS, sections were incubated with fluorescence‐conjugated secondary antibodies (Alexa Fluor 488 or 568) for 1 h at room temperature. After washing three times, sections were mounted with ProLong Gold antifade reagent with DAPI (Vector Laboratories, Burlingame, CA, USA) and visualized on a fluorescence microscope.

### RT‐PCR and quantitative RT‐PCR (qRT‐PCR)

Total RNA samples from GFP^+^ or GFP^−^ cells at E12, E16, and hatch were extracted using the ReliaPrep RNA Miniprep Systems (Promega, Madison, WI, USA). Total RNA samples were then reverse transcribed into cDNAs using the SuperScript III Reverse Transcription Kit (Invitrogen). The cDNAs were amplified by PCR using specific primer sets (30 cycles at 95 °C for 30 s, 60 °C for 30 s, and 72 °C for 30 s). Quantitative RT‐PCR (qRT‐PCR) was performed to measure the expression of genes involved in the identified pathways in germ cells and somatic gonadal cells, respectively. qRT‐PCR was performed in triplicate using a StepOnePlus RT‐PCR system (Applied Biosystems, Foster City, CA, USA). These reactions followed thermocycling conditions: 5 min at 95 °C; 40 cycles of 30 s at 95 °C, 30 s at 60 °C, and 30 s at 72 °C; and at the melting temperatures. Relative gene expression was calculated using the following formula: *DCt* = *Ct* of the target gene – *Ct* of *GAPDH*. Primer sequences are listed in Table S1.

### Statistical analysis

Statistical analysis was performed using graphpad prism 9 (GraphPad Software, San Diego, CA, USA). Significant differences between groups were determined by one‐way ANOVA with Tukey's multiple comparisons. Statistical significance was ranked as *P < 0.05 and **P < 0.01.

## Results

### Identification of mitotic‐arrested prospermatogonia and dynamic signaling pathways after initiation of mitotic arrest

**Fig. 1:**
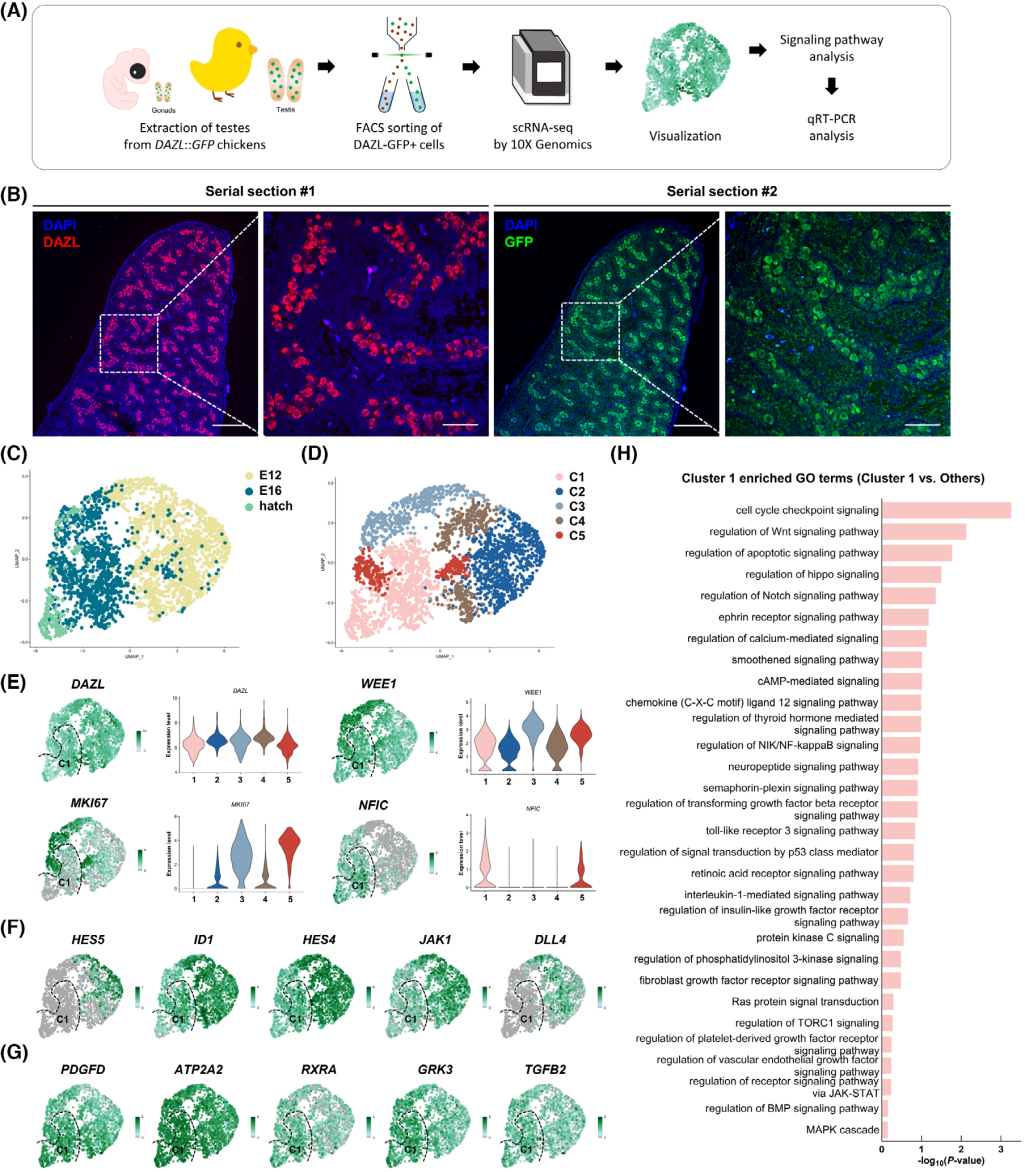
Analysis of mitotic‐arrested prospermatogonia‐specific gene expression and signaling pathway‐related gene ontology (GO) terms. (A) Illustration of experimental workflow. (B) Validation of endogenous DAZL expression in testis of germ‐cell tracing chicken models at hatching day by immunohistochemistry for DAZL and GFP in serial sections. As secondary antibodies, anti‐rabbit IgG‐Alexa 568 antibody and anti‐rabbit IgG‐Alexa 488 antibody were used, respectively. Scale bars, 200 and 50 μm (enlarged images). (C) Uniform manifold approximation and projection (UMAP) plot of chicken male germ cells from germ‐cell tracing chicken models. (D) UMAP plot visualizing the five clusters by using unsupervised clustering. (E) UMAP plots and violin plots for each cluster showing the expression levels of a germ‐cell‐specific marker (*DAZL*), proliferation‐related genes (*MKI67* and *WEE1*), and a mitotic‐arrested germ‐cell marker (*NFIC*). (F) UMAPs indicating the expression levels of top DEGs included in downregulated signaling pathways in mitotic‐arrested prospermatogonia. (G) UMAPs indicating the expression levels of top DEGs included in upregulated signaling pathways in mitotic‐arrested prospermatogonia. Top DEGs mean the genes that are ranked in the top lost when the genes are sorted in descending order of the absolute value of log_2_FC. (H) Gene Ontology enrichment (signaling pathways in biological process) analysis for cluster 1 by using the PANTHER database.

We first validated the expression of GFP and the normal expression of DAZL in the germ cells of *DAZL*::*GFP* chicks (Fig. 1A,B). To identify mitotic germ cells and mitotic‐arrested prospermatogonia, we used scRNA‐seq data of germ cells from male gonads at E12, E16, and hatch chicks [13]. We visualized results in the space calculated by using uniform manifold approximation and projection (UMAP) (Fig. 1C). We identified 5 clusters of cells according to gene expression profiles (Fig. 1D). All clusters robustly expressed a germ‐cell marker *DAZL* (Fig. 1E) [5]. Cells in clusters 3 and 5 robustly expressed a proliferation‐marker gene *MKI67* during mitotic entry at both E16 and hatch, indicating these cells are mitotic germ cells. Cells in cluster 1 corresponding to Fig. 1E expressed lower levels of *MKI67* and *WEE1* than did cells in other clusters; these genes are minimally expressed in mitotically arrested PGCs [9]. However, cluster 1 cells robustly expressed *NFIC*, a co‐marker of mitotic‐arrested germ cells in both humans and mice [9]. Therefore, we considered cluster 1 to represent nonproliferating mitotic‐arrested prospermatogonia (Fig. 1E). To confirm transcriptional changes in signaling pathway‐related genes, we identified differentially expressed genes (DEGs) whose levels in cluster 1 were higher or lower than those in other clusters (Table S2). Among cluster 1 DEGs, we found that downregulated genes had higher absolute log_2_FC values than upregulated genes (Tables S3 and S4). Expression of *HES5*, *ID1*, *HES4*, *JAK1*, and *DLL4* was drastically downregulated, and the expression of *PDGFD*, *ATP2A2*, *RXRA*, *GRK3*, and *TGFB2* was upregulated in cluster 1 (Fig. 1F,G, respectively). Next, we performed enriched gene ontology (GO) terms analysis by using the PANTHER database. Among cluster 1‐enriched GO terms, we extracted only signaling pathway‐related terms to identify changes in signal transduction involved in the transition of mitotic germ cells into mitotic‐arrested prospermatogonia (Fig. 1H and Table S5). We confirmed that many signaling pathways such as cell‐cycle checkpoint, smoothened (also known as Hedgehog), thyroid‐hormone mediated, TGF‐β, FGF, JAK–STAT, BMP, and MAPK were altered in mitotic‐arrested prospermatogonia. We therefore hypothesized that multiple signaling pathways might be dynamically involved in the mitotic arrest in male chicken germ cells.

### Expression changes in signaling pathway‐related genes after mitotic arrest in chicken male germ cells

**Fig. 2:**
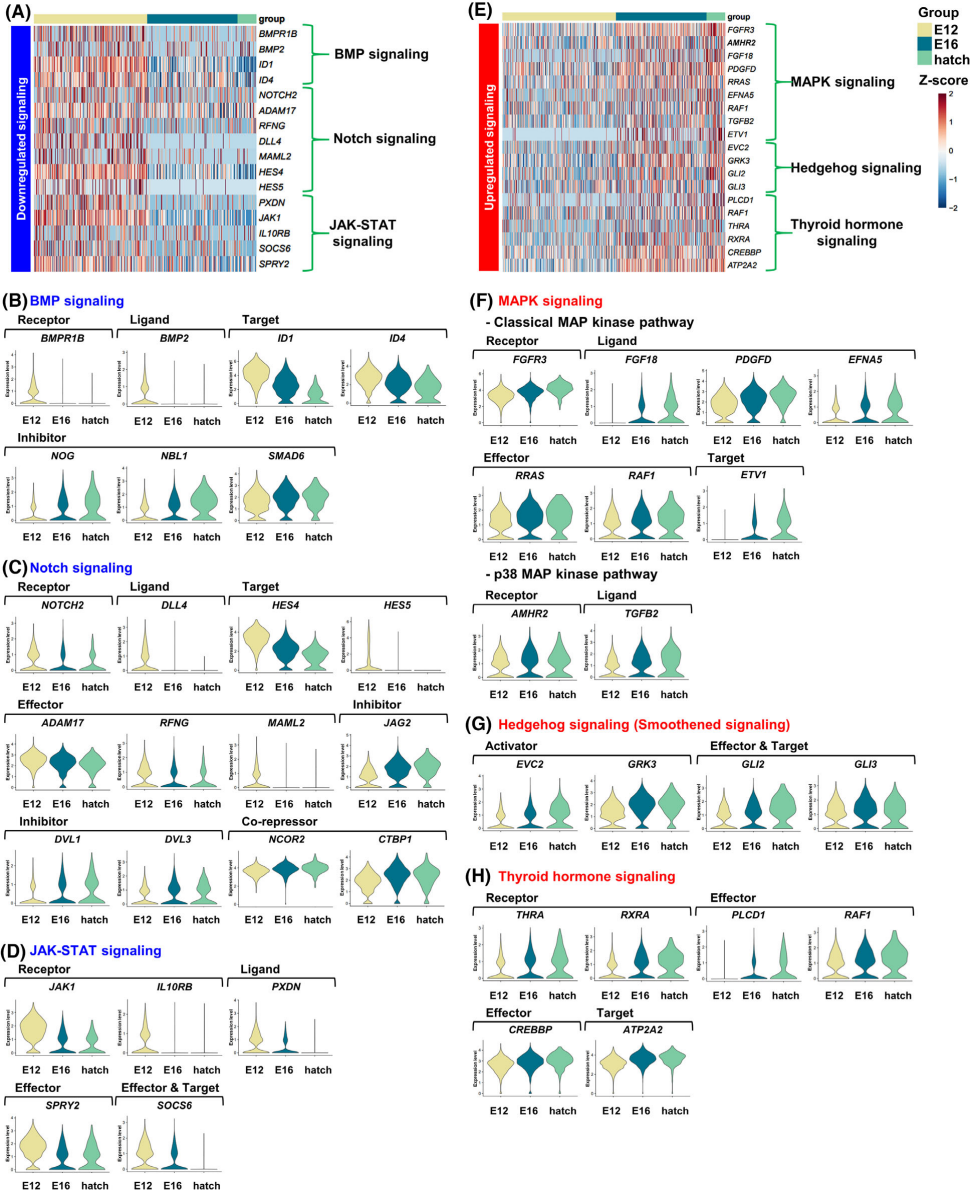
Downregulated and upregulated signaling pathways in mitotic‐arrested prospermatogonia. (A) Heatmap of downregulated signaling pathway‐related genes at E12, E16, and hatch. (B–D) Violin plots showing expression levels of BMP, Notch, and JAK‐STAT signaling‐related genes at E12, E16, and hatch. (E) Heatmap of upregulated signaling pathways‐related genes at E12, E16, and hatch. (F–H) Violin plots showing the expression levels of MAPK‐, Hedgehog‐, and thyroid hormone signaling‐related genes at E12, E16, and hatch.

We screened the expression of genes encoding receptors, ligands, effectors, activators, targets, and inhibitors of canonical signaling pathways. We found that genes related to BMP, Notch, and JAK–STAT signaling were downregulated after mitotic arrest (Fig. 2A). Genes related to the BMP‐signaling pathway including receptor *BMPR1*, ligand *BMP2* and targets *ID1* and *ID4* were downregulated from E12 to hatch. On the other hand, inhibitors *NOG*, *NBL1*, and *SMAD6* were upregulated after initiation of mitotic arrest (Fig. 2B). In the Notch‐signaling pathway, receptor *NOTCH2*, ligand *DLL4*, effectors *ADAM17*, *RFNG*, and *MAML2*, and targets *HES4* and *HES5* were downregulated from E12 to hatch. On the other hand, the genes coding for the inhibitors *JAG2*, *DVL1*, and *DVL3*, and co‐repressors *NCOR2* and *CTBP1* were progressively upregulated (Fig. 2C). In the JAK–STAT signaling pathway, receptors *JAK1* and *IL10RB*, ligand *PXDN*, effectors *SPRY2*, and target *SOCS6* were downregulated after E12 (Fig. 2D).

We also found that gene sets for several signaling pathways were preferentially expressed after mitotic arrest (Fig. 2E). In the classic MAP‐kinase pathway, receptor *FGFR3*, ligands *FGF18*, *PDGFD*, and *EFNA5*, effectors *RRAS* and *RAF1*, and target *ETV1* were upregulated from E12 to hatch (Fig. 2F). In the p38 MAP‐kinase pathway, receptor and ligand genes *AMHR2* and *TGFB2*, respectively, were upregulated from E12 to hatch (Fig. 2F). In the Hedgehog‐signaling pathway, activator genes (*EVC2* and *GRK3*) and dual‐function effector‐and‐target genes *GLI2* and *GLI3* were upregulated after mitotic arrest (Fig. 2G). Hedgehog‐pathway activation requires both ligand expression and *PTCH1*‐receptor repression [17]. However, three ligand genes (*DHH*, *IHH*, and *SHH*) were virtually unexpressed in GFP^+^ germ cells (Fig. S1A). Hence, we measured the expression of one of these ligands DHH secreted from somatic gonadal cells. We selected the DHH, because it is expressed in mouse Sertoli cells [18] and regulates germ‐cell differentiation [17]. By using RT‐PCR, we confirmed that GFP^−^ somatic gonadal cells at E12, E16, and hatch expressed *DHH* (Fig. S1B). This suggested that the DHH ligand secreted from somatic gonadal cells during mitotic arrest activates Hedgehog signaling in germ cells. We also found that thyroid‐hormone signaling pathway‐related genes coding for receptors *THRA* and *RXRA*, effectors *PLCD1* and *RAF1*, and targets *CREBBP* and *ATP2A2* progressively upregulated from E12 to hatch (Fig. 2H). All these genes demonstrated the highest expression in cluster 1, which corresponds to mitotically arrested cells. This suggests that their related pathways may be involved in the quiescent state of male germ cells.

### Downregulated signaling pathways in mitotic‐arrested prospermatogonia appeared to be suppressed after transient activation

**Fig. 3:**
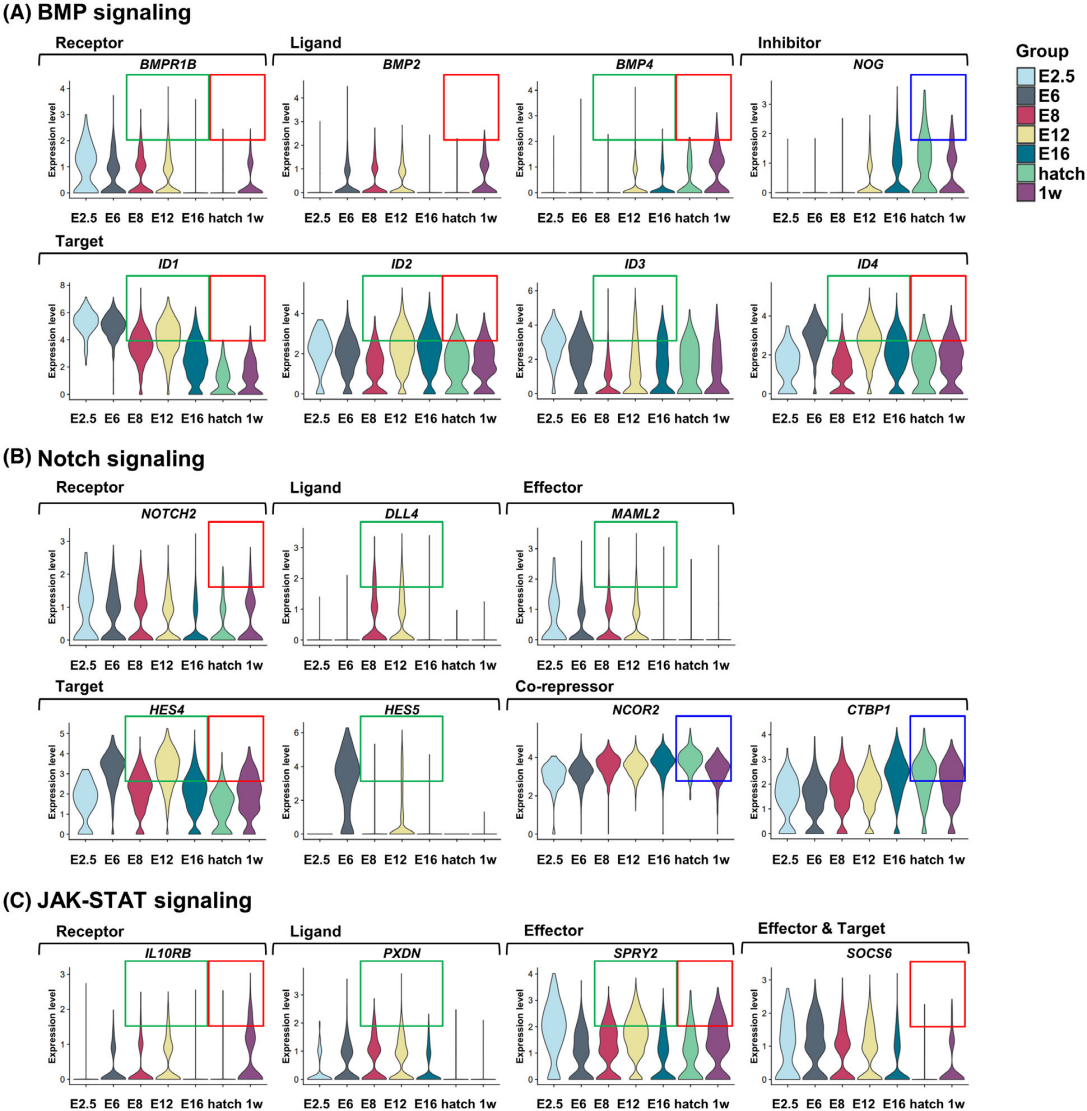
Temporal dynamic of the expression pattern of three signaling pathways during embryonic development and 1 week after hatch analyzed by single‐cell RNA sequencing. (A) Violin plots illustrating expression of BMP‐signaling‐related genes at E2.5, E6, E8, E12, E16, hatch, and 1 week after hatch. (B) Violin plots illustrating expression of Notch‐signaling‐related genes. (C) Violin plots illustrating expression of JAK‐STAT signaling‐related genes. Boxes indicate differences between mean values of normalized gene expression: Red boxes indicate upregulated expression; blue boxes, downregulated expression; and green boxes, downregulated expression after upregulated expression. w, week posthatch.

In order to investigate more detailed expression patterns of identified signaling pathway‐related genes, we performed additional analysis on five time points: E8, E12 (before the onset of mitotic arrest), E16 (entering asynchronously into mitotic arrest), hatch (mostly mitotic arrest in G0/G1), and 1 week after hatch (maintaining mitotic arrest in G0/G1). This analysis confirmed the expression profiles identified by single‐cell transcriptomic analysis for selected pathway‐related genes. Among these genes, several genes were expressed at higher levels at E12 than at E8 and gradually downregulated from E12 to hatch. This expression pattern was found for the genes involved in the activation of the BMP‐signaling pathway (such as *BMPR1B*, *BMP4*, *ID1*, *ID2*, *ID3*, and *ID4*; Fig. 3A), Notch‐signaling pathway (*DLL4*, *MAML2*, *HES4*, and *HES5*; Fig. 3B), and JAK–STAT signaling pathway (*IL10RB*, *PXDN*, and *SPRY2*; Fig. 3C). We speculate that activation of these pathways may be required for mitotic‐arrest initiation. The genes related to these pathways were next downregulated and upregulated again 1 week after hatch (Table S6). Among genes related to the BMP‐signaling pathway, the expression was upregulated in the case of *BMPR1B*, *BMP2*, *BMP4*, *ID1*, *ID2*, and *ID4*, whereas that of gene encoding BMP antagonist Noggin (NOG) downregulated 1 week after hatch [19] (Fig. 3A). Among genes related to the Notch‐signaling pathway, the expression of *NOTCH2* and *HES4* downregulated at E16 and upregulated again at 1 week after hatch, while expression of the co‐repressors *NCOR2* and *CTBP1* downregulated at the same time (Fig. 3B). The expression pattern of genes related to the JAK–STAT pathway was similar: *IL10RB*, *SPRY2*, and *SOCS6* expression downregulated at E16 and next upregulated again 1 week after hatch (Fig. 3C). Thus, the expression of genes related to BMP, NOTCH, and JAK–STAT pathways peaked at E12 (just before mitotic arrest), downregulated at E16, then upregulated again 1 week after hatch. We therefore suggest that transient changes in these signaling pathways may induce male germ cells to enter mitotic arrest.

### Upregulated signaling pathways increase steadily during mitotic arrest

**Fig. 4:**
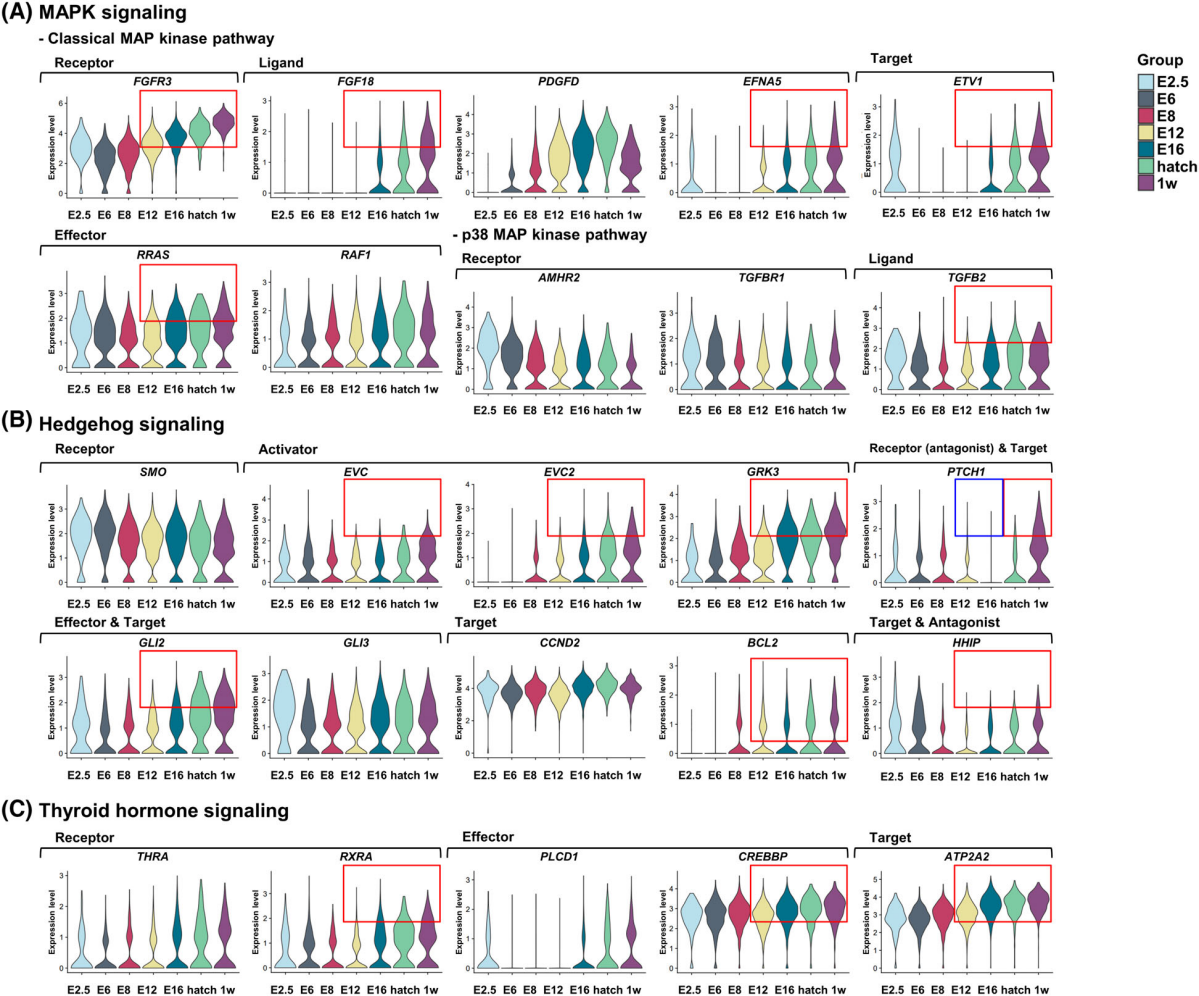
Upregulated signaling pathways in mitotic‐arrested prospermatogonia from embryonic development to 1 week after hatch. (A) Violin plots illustrating expression of MAPK‐signaling‐related genes at E2.5, E6, E8, E12, E16, hatch, and 1 week after hatch. (B) Violin plots illustrating expression of Hedgehog‐signaling‐related genes in male germ cells. (C) Violin plots illustrating expression of thyroid hormone signaling‐related genes. Boxes indicate differences between mean values of normalized gene expression: Red boxes indicate upregulated expression; blue boxes, downregulated expression. w, week posthatch.

We examined the expression patterns of genes involved in MAPK, Hedgehog, and thyroid‐hormone signaling. Expression levels of these genes were similar between E8 and E12 except for *PDGFD*. Expression of many genes upregulated after mitotic arrest and persisted after hatch. The expression of genes involved in MAPK pathway including *FGFR3*, *FGF18*, *EFNA5*, *ETV1*, *RRAS*, and *TGFB2*, in Hedgehog‐signaling pathway including *EVC*, *EVC2*, *GRK3*, *GLI2*, and *BCL2*, and in thyroid‐hormone signaling pathway including *RXRA*, *CREBBP*, and *ATP2A2* upregulated steadily from E12 to 1 week after hatch (Fig. 4A–C, respectively). *PTCH1* and *HHIP* are both targets and antagonists of the Hedgehog‐signaling pathway. *PTCH1* levels downregulated at E12–E16, the period of mitotic‐arrest transition, and upregulated significantly at 1 week after hatch, whereas *HHIP* gradually upregulated until 1 week after hatch (Fig. 4B). Considering these collective results, we propose that these upregulated signaling pathways may be involved in both mitotic arrest and subsequent quiescent state of male germ cells.

### Interactions among germ cells and somatic gonadal cells regulate BMP‐, Notch‐, and MAPK‐signaling pathways

**Fig. 5:**
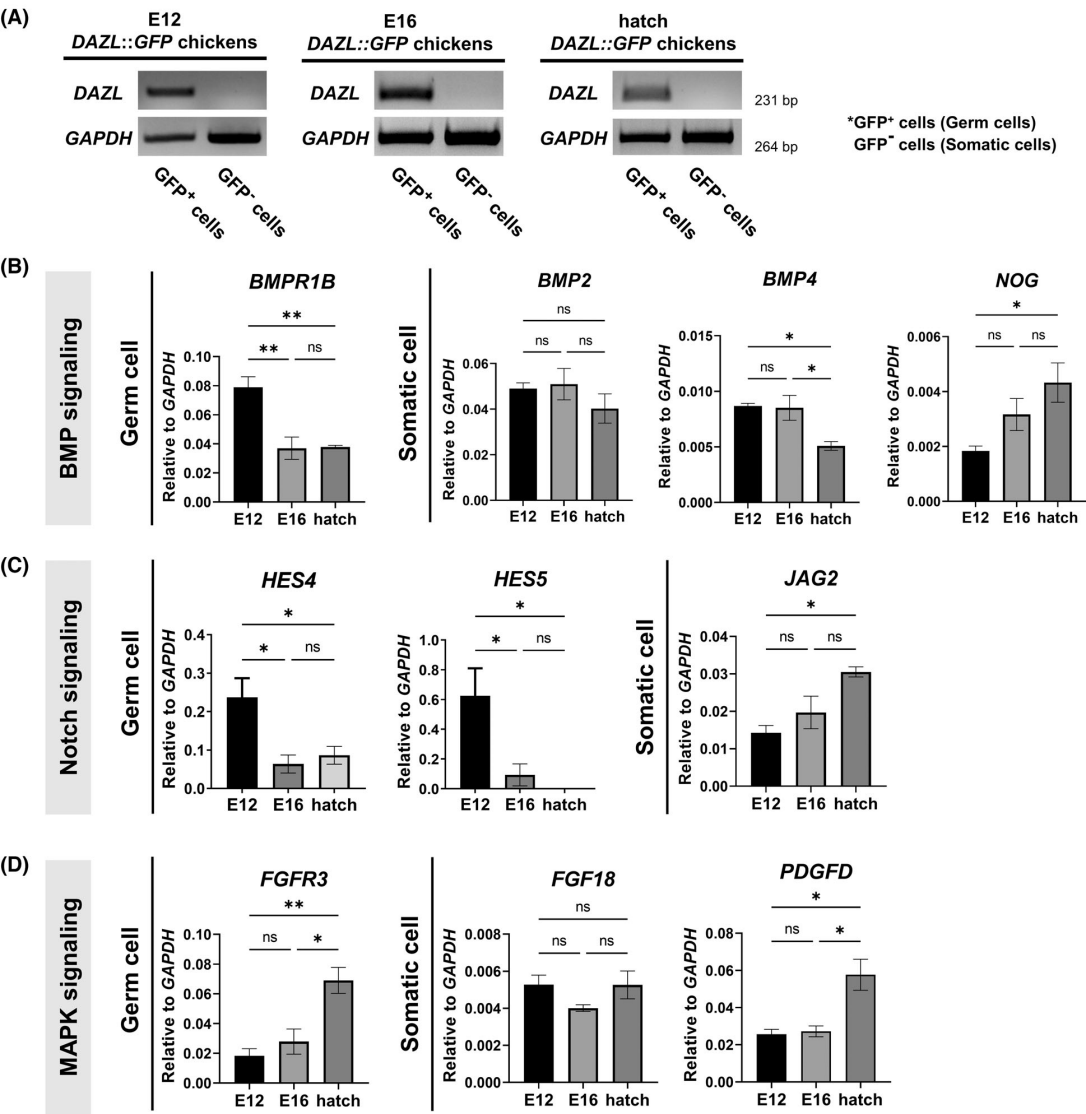
Interaction between germ cells and somatic gonadal cells for signaling pathways during mitotic arrest. (A) RT‐PCR analysis of *GAPDH* and *DAZL* in GFP^+^ and GFP^−^ cells isolated from testes of germ‐cell tracing chicken models at E12, E16, and hatch. (B) Relative expression levels of *BMPR1B* (a BMP‐signaling receptor gene) in GFP^+^ cells (germ cells) at the indicated stages. Relative expression levels of *BMP2* and *BMP4* (BMP‐signaling ligand genes) and *NOG* (a BMP‐signaling antagonist gene) in GFP^−^ cells (somatic gonadal cells). (C) Relative expression of *HES4* and *HES5* (Notch‐signaling target genes) in GFP^+^ cells. Relative expression of *JAG2* (a Notch‐signaling inhibitor gene) in GFP^−^ cells. (D) Relative expression of *FGFR3* (a receptor gene of MAPK signaling) in GFP^+^ cells. Relative expression of *FGF18* and *PDGFD* (MAPK‐signaling ligand genes) in GFP^−^ cells. Expression is measured by using qRT‐PCR, normalized to *GAPDH* expression, and presented as the mean ± standard error of mean (*n* = 3). Significant differences are determined by one‐way ANOVA with the Tukey's multiple comparison test. *P < 0.05, **P < 0.01, and ns, not significant.

Germ cells develop in interaction with surrounding somatic cells [20]. We thus investigated the role of somatic gonadal cells in the signaling pathway regulation of mitotic arrest. We selected BMP‐, Notch‐, and MAPK‐signaling pathways as representative pathways, and we isolated GFP^+^ cells (germ cells) and GFP^−^ cells (somatic gonadal cells) from testes of germ‐cell tracing chicken models [13] by FACS at E12, E16, and hatch. After using RT‐PCR to confirm *DAZL*‐expression levels in GFP^+^ germ cells (Fig. 5A), we used qRT‐PCR to measure the expression of genes involved in these pathways. We found that the expression of *BMPR1B*, a receptor of BMP signaling, downregulated in germ cells after E12. Expression of *BMP4*, a ligand of BMP signaling, downregulated in somatic gonadal cells from E12 to hatch. Expression of *NOG*, an antagonist of BMP signaling, upregulated in somatic gonadal cells from E12 to hatch (Fig. 5B). Expression of Notch‐pathway target genes *HES4* and *HES5* significantly downregulated in germ cells after E12, and expression of the inhibitor gene *JAG2* upregulated in somatic gonadal cells from E12 to hatch (Fig. 5C). Expression of FGFR3, a receptor gene of MAPK pathway, upregulated in germ cells after hatch. Also, the expression of PDGFD, one of the ligand genes in MAPK pathway, upregulated in somatic gonadal cells after hatch (Fig. 5D). *BMP2* and *FGF18* ligand genes were also expressed in somatic cells, although the expression of these genes did not significantly differ among the three time points (Fig. 5B,D). These results indicate that changes in the expression of signal molecules related to BMP, Notch, and MAPK pathways in the somatic gonadal cells may be involved in the regulation of mitotic arrest in male germ cells.

## Discussion

Male germ‐cell differentiation is controlled by various signal transduction and transcription factors and strongly dependent on surrounding somatic gonadal cells after their sex differentiation [3, 4]. Although various signaling pathways are involved in the development of chicken germ cells [11, 21], studies on the mechanisms regulating mitotically arrested male germ cells are lacking [22]. In this study, we identified signaling pathways involved in mitotic arrest and subsequent quiescent state of male germ cells by single‐cell transcriptome analysis of germ cells isolated from a germ‐cell tracing chicken model.

Stem cell differentiation requires the downregulation of genes essential for self‐renewal. For example, the downregulation of *Hes*‐family genes regulates proper neural stem‐cell differentiation [23]. BMP‐, NODAL‐, and Notch‐signaling pathways occur in mitotically arrested human fetal germ cells, and these signaling pathways are regulated by interaction with somatic cells [10]. In the mouse PGCs, the Notch‐signaling pathway is activated in mitotically arrested cells, and NOTCH1 expression increases from E12.5 (just before the onset of mitotic arrest) and downregulated at E16.5. In these PGCs, the expression of the target *HES1* gradually increases from E13.5 to E16.5. Blocking Notch signaling increases the number of MKI67‐positive cells, suggesting that Notch signaling is essential for regulating mitotic arrest in male PGCs [9]. We found lower *HES4* and *HES5* expression levels in mitotic‐arrested prospermatogonia than in mitotic cells. Thus, changes in Notch‐signaling pathway in mitotic‐arrested cells in chicken male embryonic germ cells differed from those in human and mouse. Many genes related to BMP‐, Notch‐, and JAK‐STAT signaling pathways were suppressed after initiation of mitotic arrest. We propose that these three signaling pathways, which were transiently upregulated before mitotic arrest and subsequently downregulated, may be involved in mitotic arrest in male chicken germ cells.

We found MAPK, Hedgehog, and thyroid‐hormone pathways were continuously upregulated in male germ cells during mitotic arrest. In mice, the transition from gonocyte to spermatogonia begins at E18.5. Moreover, FGF signaling prominently regulates pathways upstream of GDNF, retinoic acid, MEK/ERK, and PI3K/Akt signaling [1]. In addition, the main function of *Fgf9* expressed in both somatic and germ cells in mice is to repress female‐promoting genes [24], and p38‐MAPK signaling also represses retinoic acid signaling [25]. Thus, pathways upregulated after mitotic arrest (including the MAPK pathway) may facilitate the early development of prospermatogonia into spermatogonia and inhibit the female‐specific pathway. In mice and rats, ligand (such as Dhh) secreted from Sertoli cells activates Hedgehog signaling in Leydig cells, which regulates testis development and germ‐cell survival [26, 27]. Thus, Hedgehog‐signaling activation in mitotic‐arrested chicken prospermatogonia may be important for the survival and differentiation of germ cells and may be a central signal that regulates mitotic‐arrest initiation because upregulated expression of *PTCH1* and *HHIP* activates negative feedback. Thyroid hormone is involved in testis development and spermatogenesis in several vertebrates [28] and in the development of prospermatogonia in mice [12]. Thyroid hormones, especially T_4_ activates ERK1/2 to initiate activities of TRβ1, ERα, and STAT, and these hormones regulate transcriptional activities, cytokines, and growth factors [29]. We speculate that the thyroid‐hormone‐related genes activated in mitotic‐arrested prospermatogonia of chickens may be important for activating MAPK/ERK signals and promoting differentiation, but further research is required.

## Conclusion

In this study, we identified changes in the gene expression profile of components of BMP‐, Notch‐, JAK‐STAT, MAPK, Hedgehog, and thyroid‐hormone signaling pathways before, during, and after mitotic‐arrest transition in male chicken germ cells. We propose that these pathways are involved in mitotic arrest of male germ cells during embryonic development and subsequent quiescent state after hatching. Signaling pathways that are upregulated before the initiation of mitotic arrest, and downregulated thereafter, may initiate mitotic arrest and male‐specific development. Overall, our findings give insight into the regulation of signaling pathways involved in different steps of mitotic arrest of male germ cells in chickens.

## Conflict of interest

The authors declare no conflict of interest.

### Peer review

The peer review history for this article is available at https://publons.com/publon/10.1002/2211‐5463.13600.

## Author contributions

JYH conceived and supervised the study. HJC and KMJ designed the study and prepared the manuscript. HJC and KMJ carried out single‐cell RNA‐seq data analysis and performed the experiments. KMJ made manuscript revisions. KJP, KYL, and SJW assisted in animal management. All authors have read and approved the final manuscript.

## Supporting information


**Fig. S1.** Confirmation of ligand genes for Hedgehog signaling in DAZL::GFP‐positive cells and DAZL::GFP‐negative cells during mitotic arrest.

**Table S1.** List of primers used for research.
**Table S3.** List of top 5 DEGs included in downregulated signaling pathways when DEGs of cluster 1 are sorted in descending order by the absolute value of log_2_FC.
**Table S4.** List of top 5 DEGs included in upregulated signaling pathways when DEGs of cluster 1 are sorted in descending order by the absolute value of log_2_FC.
**Table S6.** List of genes whose expression was changed at 1 week after hatch compared with hatch in downregulated signaling pathways. One week after hatch/hatch represents the mean value of 1 week after hatch divided by the mean value of hatch for each normalized gene expression.

**Table S2.** List of DEGs in cluster 1 (Cluster 1 vs others).

**Table S5.** List of enriched GO terms‐related signaling pathways in cluster 1.

## Data Availability

The scRNA‐seq data have been deposited in the SRA database (PRJNA761874). Other datasets generated during and/or analyzed during the current study are available upon request.
