# Supplementary material for: Single‐cell transcriptome analysis of male chicken germ cells reveals changes in signaling pathway‐related gene expression profiles during mitotic arrest
Source: FEBS Open Bio. 2023 Mar 30;13(5):833–44. doi: 10.1002/2211-5463.13600 (PMC10153307; doi:10.1002/2211-5463.13600)
Supplement: Supplementary file 1 — Fig. S1. Confirmation of ligand genes for Hedgehog signaling in DAZL::GFP‐positive cells and DAZL::GFP‐negative cells during mitotic arrest. [file FEB4-13-833-s001.docx]

**
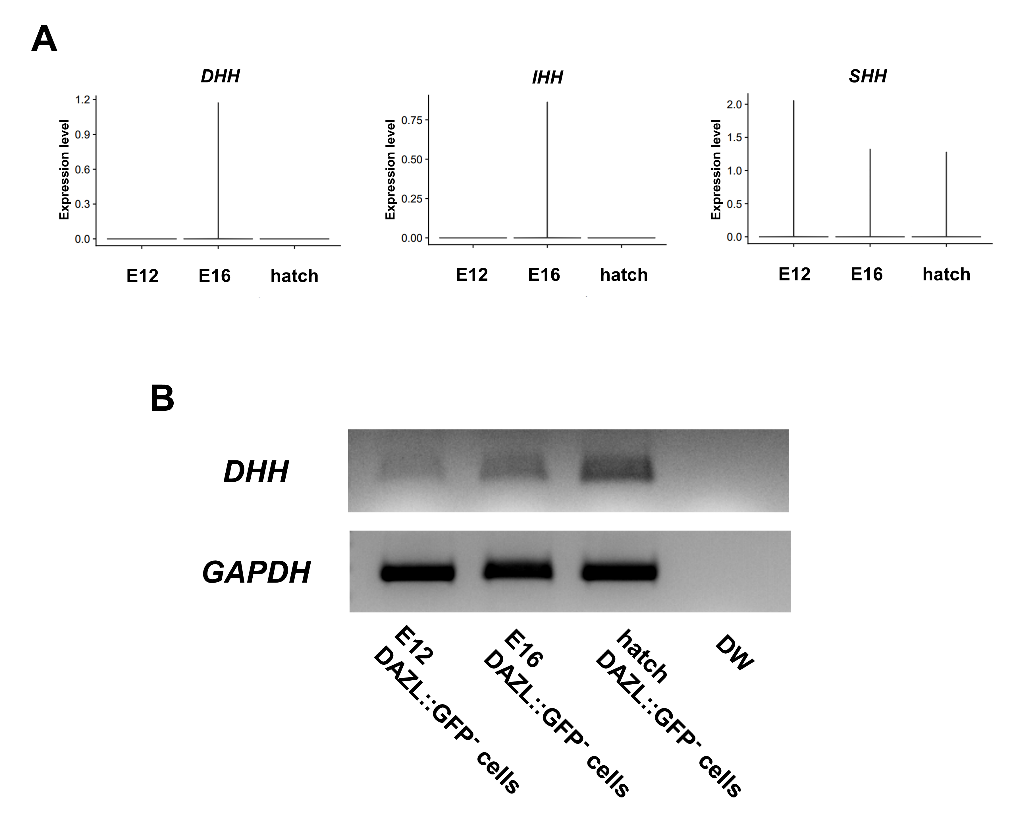
**

**Fig. S1.** Confirmation of ligand genes for Hedgehog signaling in DAZL::GFP-positive cells and DAZL::GFP-negative cells during mitotic arrest. (A) Violin plots indicating the expression level of ligand encoding genes of Hedgehog signaling at E12, E16, and hatch. (B) RT-PCR analysis of *DHH* and *GAPDH* in DAZL::GFP-negative cells isolated from testes of *DAZL::GFP* chickens at E12, E16, and hatch.
