# Supplementary material for: Single‐cell transcriptome analysis of male chicken germ cells reveals changes in signaling pathway‐related gene expression profiles during mitotic arrest
Source: FEBS Open Bio. 2023 Mar 30;13(5):833–44. doi: 10.1002/2211-5463.13600 (PMC10153307; doi:10.1002/2211-5463.13600)
Supplement: Supplementary file 2 — Table S1. List of primers used for research. Table S3. List of top 5 DEGs included in downregulated signaling pathways when DEGs of cluster 1 are sorted in descending order by the absolute value of log2FC. Table S4. List of top 5 DEGs included in upregulated signaling pathways when DEGs of cluster 1 are sorted in descending order by the absolute value of log2FC. Table S6. List of genes whose expression was changed at 1 week after hatch compared with hatch in downregulated signaling pathways. One week after hatch/hatch represents the mean value of 1 week after hatch divided by the mean value of hatch for each normalized gene expression. [file FEB4-13-833-s003.docx]

**Table S1.** List of primers used for research.

| **Gene symbol** | **Description** | **Primer sequence (5’→3’)** | | **Usages** |
| --- | --- | --- | --- | --- |
| *GAPDH* | glyceraldehyde-3-phosphate dehydrogenase | F: | GGT GGT GCT AAG CGT GTT AT | RT-PCR,  qRT-PCR |
|  |  | R: | ACC TCT GCC ATC TCT CCA CA |  |
| *DAZL* | deleted in azoospermia like | F: | CAA CTA TCA GGC TCC ACC AC | RT-PCR |
|  |  | R: | CTC AGA CGG TTT TCA GGG TT |  |
| *DHH* | desert hedgehog | F: | TGA TGA ATA TGT GGC CGG GG | RT-PCR |
|  |  | R: | ATG TGC GCT TTG GAC TCG TA |  |
| *BMPR1B* | bone morphogenetic protein receptor type 1B | F: | ATT AGA GGG CTC GGA CTT | qRT-PCR |
|  |  | R: | GCT TCT TGC CGC TTG |  |
| *BMP2* | bone morphogenetic protein 2 | F: | CTC AGC TCA GGC CGT TGT TA | qRT-PCR |
|  |  | R: | GTC ATT CCA CCC CAC GTC AT |  |
| *BMP4* | bone morphogenetic protein 4 | F: | CGC TGG GAG ACC TTT GAT GT | qRT-PCR |
|  |  | R: | CCC CTG AGG TAA AGA TCG GC |  |
| *NOG* | noggin | F: | AAG GGG CTG GAG TTC TAC GA | qRT-PCR |
|  |  | R: | GGT TTG CAG ACC ATG CCT TC |  |
| *HES4* | hes family bHLH transcription factor 4 | F: | GCA CCG GAA GTC CTC CAAA C | qRT-PCR |
|  |  | R: | TCA TTG AAA CCA GCC CGG TA |  |
| *HES5* | hes family bHLH transcription factor 5 | F: | AGC GCT CTC TCC CTG GAA AT | qRT-PCR |
|  |  | R: | TCC TGC TGT AGG CTT TTG GC |  |
| *JAG2* | jagged canonical Notch ligand 2 | F: | TCC CAT GGG TCA AGA ATG CC | qRT-PCR |
|  |  | R: | CCC TGG GGG ACT TTG TTT CC |  |
| *FGFR3* | fibroblast growth factor receptor 3 | F: | ACA GGT AAC AGT GTC GTT GGA | qRT-PCR |
|  |  | R: | CAA AAC AGC CCT CAC CAA GC |  |
| *FGF18* | fibroblast growth factor 18 | F: | GGC AGT CAA GTC CGG ATC AA | qRT-PCR |
|  |  | R: | TTT CCG TGG CCT CCC TTT TT |  |
| *PDGFD* | platelet derived growth factor D | F: | CGG GAT GAG AGC AAT CAC CT | qRT-PCR |
|  |  | R: | CAG GCT CCT CCA GTC CAA AC |  |

**Table S3.** List of top 5 DEGs included in downregulated signaling pathways when DEGs of cluster 1 are sorted in descending order by the absolute value of log_2_FC.

| **Gene** | ***P*-value** | **Average log_2_FC** | **Pct. 1** | **Pct. 2** | ***P*_val_adj** |
| --- | --- | --- | --- | --- | --- |
| *HES5* | 3.05598E-61 | -3.613203589 | 0.005 | 0.267 | 6.35216E-57 |
| *ID1* | 5.82672E-94 | -1.726520509 | 0.799 | 0.957 | 1.21114E-89 |
| *HES4* | 6.9026E-121 | -1.557962597 | 0.849 | 0.959 | 1.4348E-116 |
| *JAK1* | 2.8645E-108 | -1.313458155 | 0.443 | 0.802 | 5.9541E-104 |
| *DLL4* | 2.50936E-79 | -1.184948674 | 0.027 | 0.367 | 5.21595E-75 |

**Table S4.** List of top 5 DEGs included in upregulated signaling pathways when DEGs of cluster 1 are sorted in descending order by the absolute value of log_2_FC.

| **Gene** | ***P*-value** | **Average log_2_FC** | **Pct. 1** | **Pct. 2** | ***P*_val_adj** |
| --- | --- | --- | --- | --- | --- |
| *PDGFD* | 4.056E-58 | 0.8421463 | 0.928 | 0.861 | 8.431E-54 |
| *ATP2A2* | 5.549E-86 | 0.6961396 | 0.995 | 0.993 | 1.153E-81 |
| *RXRA* | 1.852E-36 | 0.6684969 | 0.669 | 0.504 | 3.849E-32 |
| *GRK3* | 6.903E-36 | 0.6170325 | 0.881 | 0.82 | 1.435E-31 |
| *TGFB2* | 2.963E-12 | 0.5177813 | 0.691 | 0.64 | 6.158E-08 |

**Table S6.** List of genes whose expression was changed at 1 week after hatch compared to hatch in downregulated signaling pathways. 1 week after hatch/hatch represents mean value of 1week after hatch divided by mean value of hatch for each normalized gene expression.

| **Gene** | **Related signaling pathway** | **1 week after hatch/hatch** |
| --- | --- | --- |
| *BMPR1B* | BMP | 1.596026 |
| *BMP2* | BMP | 2.01662 |
| *BMP4* | BMP | 2.313887 |
| *ID1* | BMP | 1.712479 |
| *ID2* | BMP | 1.159062 |
| *ID4* | BMP | 1.230796 |
| *NOTCH2* | Notch | 1.787275 |
| *HES4* | Notch | 2.370275 |
| *IL10RB* | JAK-STAT | 2.016987 |
| *SPRY2* | JAK-STAT | 1.588486 |
| *SOCS6* | JAK-STAT | 1.732691 |
| *NOG* | BMP | 0.520722 |
| *NCOR2* | Notch | 0.580598 |
| *CTBP1* | Notch | 0.794348 |
